# Development and validation of a HPTLC method for Estimation of Duloxetine Hydrochloride in Bulk Drug and in Tablet Dosage Form

**DOI:** 10.4103/0250-474X.41463

**Published:** 2008

**Authors:** Suneela S. Dhaneshwar, P. Deshpande, M. Patil, G. Vadnerkar, S. R. Dhaneshwar

**Affiliations:** Department of Pharmaceutical Chemistry, Poona College of Pharmacy, Bharati Vidyapeeth University, Erandwane, Pune-411 038, India

**Keywords:** Duloxetine hydrochloride, HPTLC, densitometric estimation, method development and validation

## Abstract

Duloxetine hydrochloride is a potent dual reuptake inhibitor of serotonin and norepinephrine used to treat major depressive disorders. The present work describes a simple, precise and accurate HPTLC method for its estimation as bulk and in tablet dosage form. The chromatographic separation was carried out on precoated silica gel 60 F254 aluminium plates using mixture of chloroform:methanol (8:1 v/v) as mobile phase and densitometric evaluation of spots was carried out at 235 nm using Camag TLC Scanner-3 with win CAT 1.3.4 version software. The experimental parameters like band size of the spot applied, chamber saturation time, solvent front migration, slit width etc. were critically studied and optimum conditions were evolved. The drug was satisfactorily resolved with Rf value 0.11±0.01. The accuracy and reliability of the proposed method was ascertained by evaluating various validation parameters like linearity (40-200 ng/spot), precision (intra-day RSD 0.46-0.75%, inter-day RSD 0.46-1.59%), accuracy (98.72±0.20) and specificity according to ICH guidelines. The proposed method can analyse ten or more formulation units simultaneously on a single plate and provides a faster and cost-effective quality control tool for routine analysis of duloxetine hydrochloride as bulk drug and in tablet formulation.

Duloxetine hydrochloride (DH), chemically (+)-(S)-N-methyl-3-(1-naphthyloxy)-3-(thiophen-2-yl)-propan-1-amine is a drug that primarily targets major depressive disorders and pain related to diabetic neuropathy^1^–^3^. It is a potent dual reuptake inhibitor of serotonin and norepinephrine. Literature review reveals that several HPLC^4^–^6^ and LC-MS^7^ methods have been reported for estimation of duloxetine hydrochloride in single and combined form with other drugs, but no HPTLC method is reported so far. The present study illustrates development and validation of a simple, accurate, economical and reproducible procedure for determination of duloxetine hydrochloride by HPTLC as bulk and in tablet dosage form.

Pharmaceutical grade duloxetine hydrochloride working standard was a generous gift from Hetero Drugs Ltd. Erragadda, Hyderabad. Fixed dose tablets (Duvanta-20) containing 20 mg of duloxetine hydrochloride were procured from Intas Pharmaceuticals Ltd. Ahmedabad. Silica gel 60 F_254_ TLC plates (20×20 cm, layer thickness 0.2 mm, E. Merck, Germany) were used as stationary phase. All chemicals and reagents used were of analytical grade and were purchased from Merck Chemicals Corporation Ltd. Mumbai, India. A Camag HPTLC system containing Camag Linomat IV semiautomatic sample applicator, Hamilton syringe (100 μl), Camag TLC Scanner-3 with win CAT software version 1.3.4, Camag twin- trough chamber (20×10 cm) and Remi centrifuge (Model C30) were used for the present study.

Duloxetine hydrochloride (20 mg) was weighed accurately and transferred to 10 ml volumetric flask. The volume was made upto 10 ml with methanol to obtain concentration of 2 μg/μl. 0.1 milliliter of the above solution was further diluted with methanol to obtain the concentration 0.02 μg/μl of duloxetine hydrochloride. For analysis in tablet dosage form, twenty tablets were weighed (each containing 20 mg of DH) and their average weight was calculated. The tablets were finely powdered and powder equivalent to 20 mg of duloxetine hydrochloride was accurately weighed and dissolved in 10 ml of methanol to obtain the concentration of 2 μg/μl. The solution was centrifuged for 15 min at 600 rpm. The solution was filtered through Whatman filter paper no. 41, the residue was washed with methanol and volume was adjusted to 10 ml with the same solvent. This solution was further diluted with methanol so as to have concentration same as that of final standard solution.

TLC plates were pre-washed with methanol. Activation was done in oven at 105° for 20 min. The plates were allowed to cool at room temperature. The chromatographic estimations were performed using following conditions: stationary phase, precoated silica gel 60 F_254_ aluminium plates (20 cm×20 cm×250 μm); mobile phase, chloroform:methanol (8:1 v/v); chamber saturation time, 20 min; wavelength of scanning, 235 nm; slit dimensions, 6.00×0.30 mm; spotting parameters used were, band width, 8 mm and space between two bands, 15.4 mm.

Four microlitres of standard solution of duloxetine hydrochloride (0.02 μg/μl) was applied on pre-washed and activated plate under nitrogen stream using semiautomatic spotter. It was developed at constant temperature in a Camag twin-trough chamber previously saturated for 20 min with mobile phase. The plate was removed from the chamber and air dried. Densitometric measurements were performed at 235 nm in reflectance mode with Camag TLC Scanner 3 using win CAT software version 1.3.4 incorporating track optimization position. For the preparation of a calibration curve, aliquots of 2, 4, 6, 8, 10 μl of standard solutions of duloxetine hydrochloride (0.02 μg/μl) were applied on the TLC plate using semiautomatic spotter under nitrogen stream. TLC plates were dried, developed and densitometrically analysed as described earlier.

The method was validated as per the ICH guidelines in terms of linearity, accuracy and specificity, intra-day and inter-day precision, repeatability of measurement of peak area as well as repeatability of sample application.

Literature survey revealed that several HPLC and LC-MS methods have been reported for estimation of duloxetine hydrochloride which is sophisticated but costly and time consuming. As no HPTLC method has been reported so far for estimation of duloxetine hydrochloride, the present study was aimed at development of a versatile, speedy and cost effective HPTLC technique for determination of duloxetine hydrochloride as bulk and in tablet dosage form.

Since duloxetine hydrochloride is freely soluble in methanol, tablet powder was extracted with methanol. Centrifugation for 15 min at 600 rpm helped to completely extract the drug from tablet matrix. Various solvent systems like mixture of chloroform: methanol: acetic acid, chloroform:ethanol:formic acid, chloroform:toluene, chloroform:benzene:toluene, chloroform:benzene:toluene:acetic acid were tried to separate and resolve spot of duloxetine hydrochloride from its impurities and other excipients of formulations. The mixture of chloroform: methanol (8:1 v/v) could resolve DH spot with better peak shape (fig 1). The drug was satisfactorily resolved with R_f_ value 0.11±0.01. Pre-saturation of TLC chamber with mobile phase for 30 min assured better reproducibility in migration of duloxetine hydrochloride and better resolution.

**Fig 1:**
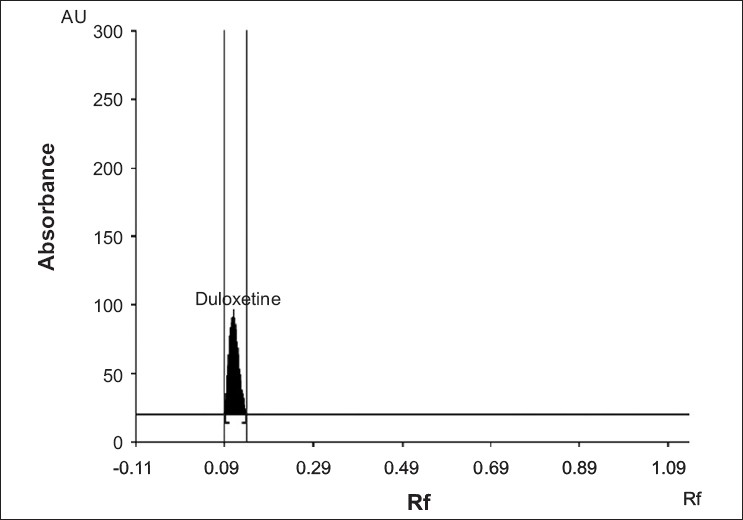
Chromatogram of duloxetine hydrochloride in chloroform: methanol (8:1 v/v).

The proposed method was validated according to ICH guidelines in terms of linearity, accuracy, inter and intra-day precision, repeatability and specificity (Table 1). The method was found to be linear in the range of 40-200 ng/spot (Y = -32.09+6.272X), (r^2^ = 0.99981) in six replicates. Accuracy of the analysis was evaluated by carrying out a recovery study at three different levels namely 80, 100, and 120 %. The results of recovery study indicate that the proposed method is accurate for estimation of drug in tablet dosage form (Table 2). The intra-day precision was determined by analyzing standard solutions of duloxetine hydrochloride in range 40-200 ng/spot for three times on the same day while inter-day precision was determined by analyzing corresponding standards on three different days over a period of one week. The intra-day and inter-day relative standard deviations were found in the range 0.46-075 % and 0.46-1.59 %, respectively. The smaller values of intra-day and inter-day variation in the analysis indicate that the method is precise.

**TABLE 1:** SUMMARY OF VALIDATION PARAMETERS

Parameters	Result of duloxetine hydrochloride
Linearity range	40-200 ng
Correlation coefficient (r)	0.99981
Standard deviation	0.70
Accuracy (n = 6)	98.72±0.20
Repeatability of sample application (n = 6)	2.08%
Repeatability of measurement of peak area ( n = 6)	0.22%
Precision	
Inter-day (n = 3)	0.46-1.59%
Intra-day (n = 3)	0.46-0.75%
Specificity	Specific

Different validation parameter of the proposed HPTLC method for estimation of duloxetine hydrochloride in tablet dosage form

**TABLE 2:** RECOVERY OF DULOXETINE HYDROCHLORIDE

Label claim^a^ (mg / tablet)	Amount added (%)	Total amount added (mg)	Amount recovered (mg)	% Recovery*	Average recovery%
Duloxetine	80	16	15.85	99.04±0.23	98.72±0.20
hydrochloride	100	20	19.70	98.69±0.24	
20	120	24	23.62	98.44±0.13	

* Average value±standard deviation of six determinations, ^a^Amount of duloxetine hydrochloride according to label claim = 20 mg, Formulation - Duvanta-20, Intas Pharmaceuticals Ltd., Ahmedabad

Repeatability of measurement of peak area was determined by spotting 6 μl of standard drug solution on TLC plate. After developing the plate, separated spot of DH was scanned six times without changing position of the plate and RSD for measurement of peak area was calculated and was found to be 0.22%.

Repeatability of sample application was assessed by spotting 6 μl of standard drug solutions six times on a TLC plate by semiautomatic spotter, followed by development of plate and recording the peak areas for six spots. The RSD for the peak area values was calculated and was found to be 2.08%. The RSD values for measurement of peak area and sample application were both below the instrumental specifications (1% and 3%, respectively), ensuring proper functioning of HPTLC system.

To confirm the specificity of the proposed method, duloxetine hydrochloride was spotted on TLC plate, developed and scanned as described earlier. It was observed that excipients present in formulation did not interfere with peak of duloxetine hydrochloride (R_f_, 0.11±0.01). The UV spectrum of standard duloxetine hydrochloride (fig. 2) was also compared with spectrum of duloxetine hydrochloride extracted from tablet, which showed good correlation. The proposed HPTLC method was found to be rapid, specific, precise and accurate.

**Fig 2:**
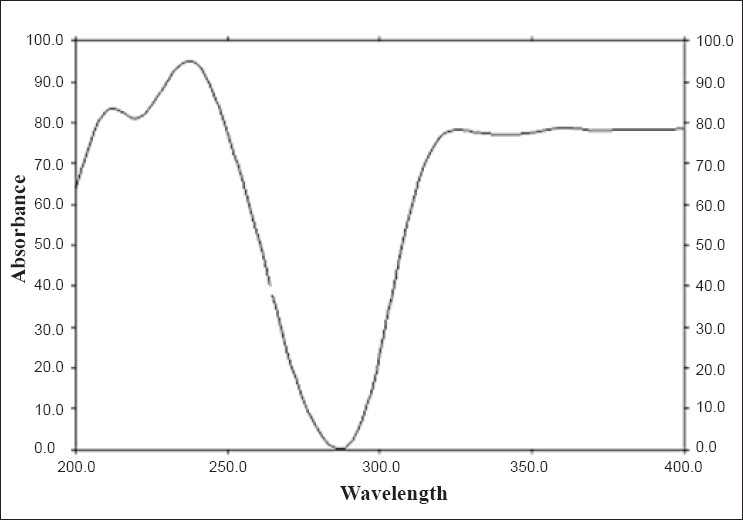
UV Spectrum of duloxetine hydrochloride in methanol.
